# Supplementary material for: Protonic nickelate device networks for spatiotemporal neuromorphic computing
Source: Nat Nanotechnol. 2026 Mar 9;21(4):579–87. doi: 10.1038/s41565-026-02133-0 (PMC13106037; doi:10.1038/s41565-026-02133-0)
Supplement: Supplementary file 1 — Supplementary Texts 1–13, Figs. 1–35, Table 1 and references. [file 41565_2026_2133_MOESM1_ESM.pdf]

nature nanotechnology

Supplementary information

https://doi.org/10.1038/s41565-026-02133-0

Protonic nickelate device networks for spatiotemporal neuromorphic computing

In the format provided by the authors and unedited

This supplementary information includes:

Supplementary Text 1 to 13

Supplementary Figs. 1 to 35

Supplementary Table 1

Supplementary References

Supplementary Text 1: Electrochemical impedance spectroscopy studies on NNO-HNNO phase transition

Electrochemical impedance spectroscopy (EIS) is a complementary technique to study the phase transition from metallic NNO to insulating H-NNO in control samples. The nature of Nyquist plots changes from nearly linear to semicircle upon metal-to-insulator phase transition in correlated oxides[1]. First, we have fabricated control devices by depositing non-catalytic Ni electrodes on NNO films. After annealing in Ar: H_2 forming gas at 115 °C for 30 minutes, we have performed room-temperature EIS experiments. The resulting Nyquist plots is shown in **Fig. S4(a)**. The Nyquist plot is an almost vertical line, indicating the conducting nature of the control sample. Then, we performed EIS experiments on devices with catalytic Pd electrodes on NNO films. The Nyquist plots show significant change before and after annealing in the forming gas at the same conditions, as shown in **Figs. S4(b)** and **S4(c)**, suggesting metal to insulator phase transition. The I-V curves in **Fig. S4(d)** taken before and after forming gas annealing shows significant increase in film resistance, supporting the formation of insulating H-NNO. In **Fig. S4(e)**, we have used the Arrhenius equation to estimate the activation energy for proton migration:

$$\log(\sigma T) = -\frac{E_a}{k_B T} + \log(\sigma_0) \quad (1)$$

where k_B is the Boltzmann's constant, and σ_0 is a constant. The slope of this graph gives the value of E_a as 0.4 eV. This value agrees well with previous reports on proton migration activation energy in H-NNO, including both experimental[2] and theoretical predictions[3].

Supplementary Text 2: Conduction Mechanisms in Pd-Au and Pd-Pd Device Structure

To investigate the current-voltage characteristic of our Pd-Au and Pd-Pd devices, we studied the changes in the energy band structure of hydrogen-doped $NdNiO_3$ (H-NNO) before and after interfacing with $NdNiO_3$ (NNO), Pd or Au. This analysis provides insights into the different DC switching behavior in Pd-Au and Pd-Pd devices. The work functions ϕ of Pd, Au, NNO and H-NNO representing the difference between vacuum level E_{vac} and Fermi level E_F are shown in **Fig. S6**[4,5]. The hydrogen doping into the pristine NNO lattice modifies the electron configuration of e_g orbital of Ni^{3+} to a strongly correlated Ni^{2+} structure leading to the metal-to-insulator transition (MIT)[6]. The opened bandgap after hydrogen doping (H-NNO) is ~3 eV[3]. Furthermore, Hall effect measurements[7,8] reveal that as NNO transitions into the insulating phase, the Hall coefficient R_H becomes electron-like. This suggests that the Fermi level in H-NNO is closer to the conductive band E_C.

For the Pd-Au device under equilibrium conditions, the band diagram of the Pd/H-NNO/NNO/Au system after contact is shown in **Fig. S7**. As illustrated in **Fig. S7**(a), due to the difference in work functions ($\phi_{NNO} > \phi_{HNNO}$), electrons transfer from H-NNO to NNO. This electron transfer creates a negative charge in NNO and a corresponding positive charge in H-NNO, forming a depletion region and a Schottky barrier at the HNNO surface. In contrast, the contact of H-NNO and Pd does not form a considerable barrier because $\phi_{Pd} > \phi_{HNNO}$. **Fig. S7**(b) provides a simplified schematic of these connections.

When a positive voltage bias is applied to Pd with Au grounded, as shown in **Fig. S8**(a), the Fermi energy of NNO becomes higher relative to H-NNO, which results in an increase of the barrier potential across the H-NNO junction that further blocks the migration of electrons from H-NNO to the NNO. Conversely, when Pd has negative voltage bias (**Fig. S8**(b)), the potential barrier across H-NNO decreases, allowing easier electron diffusion. It is consistent with our DC-sweep measurement result of the Pd-Au device, where the absolute current level under negative voltage sweep is larger than under positive bias. Moreover, the asymmetric switching behavior could be explained by the interaction between the external applied electric field and

the built-in field formed by the H-NNO/NNO Schottky barrier. When the external applied voltage bias is positive in the Pd-Au device, it has an opposite electric field direction comparing with the built-in electric field which makes the hydrogen expansion behavior driven by positive electric field from Pd electrode become harder. When it comes to negative voltage bias, the external applied electric field aligns with the built-in electric field, accelerating the hydrogen cloud moving back toward the Pd electrode, leading to a larger switching window for negative voltage sweeps compared to positive ones. The energy band diagram of the Pd-Pd device, consisting of Pd/H-NNO/NNO/H-NNO/Pd layers under voltage bias, is shown in **Fig. S12**(a). Since the Pd-Pd device is symmetric, only one voltage bias configuration is depicted. As discussed earlier in the Pd-Au device, the interface between Pd and H-NNO forms an ohmic contact without barrier, while the H-NNO/NNO interface forms a barrier. Consequently, two back-to-back Schottky junction barriers are present at two NNO/H-NNO interfaces in the Pd-Pd device as illustrated in **Fig. S12**(b). under either positive or negative voltage bias, the applied electric field decreases the barrier height at one H-NNO/NNO interface but increases it at the other, resulting in a low current. This explains the lower current level observed in Pd-Pd devices during DC-sweep compared to Pd-Au devices.

Regarding the switching behaviour, voltage bias causes protons at the two H-NNO/NNO interfaces to simultaneously migrate in the same direction under the applied electric field. This results in one hydrogen cloud expanding, which increases resistance, while the other shrinks, reducing resistance, leading to a relatively smaller switching ratio than in asymmetrical Pd-Au devices. Additionally, the barrier with increasing height consistently corresponds to hydrogen cloud expansion against the Pd electrode, while the barrier with decreasing height aligns with hydrogen cloud migration back toward the Pd. This asymmetry facilitates resistance decrease over resistance increase, explaining the small SET window (overall resistance decrease) observed under both positive and negative voltage sweeps in the Pd-Pd device.

Supplementary Text 3: Kelvin probe force microscopy (KPFM) characterization of hydrogen cloud movement

Kelvin Probe Force Microscopy (KPFM) studies on Pd-Au and Pd-Pd devices are presented in **Fig. S13**, visualizing the movement of hydrogen clouds under an electric field in nickelate devices. The changes in contact potential difference (V_{CPD}) correspond directly to the redistribution of protons across the device surface. For Pd-Au devices, after applying 100 pulses of +5V with 500 ns pulse width at the Pd electrode (with the Au electrode grounded), a proton-rich region forms at the edge of the Pd electrode. This leads to a higher V_{CPD} peak on the right side of the graphs[9]. The average width of the hydrogen cloud expands from ~2.95 μm to ~3.08 μm, reflecting the unidirectional migration of protons toward the Au electrode. In the Pd-Pd structure, two proton-rich regions form at the edges of both Pd electrodes. After applying the same pulse configuration, the proton cloud at one electrode edge expands from ~2.25 μm to ~2.65 μm, while the cloud at the opposite edge shrinks from ~2.62 μm to ~2.47 μm. This demonstrates the bidirectional migration of hydrogen ions characteristic of Pd-Pd devices, emphasizing the competing hydrogen cloud dynamics at both electrodes.

Supplementary Text 4: Investigation of temporal characteristics in Pd-Au and Pd-Pd devices

Short-term memory characteristics are observed in Pd-Pd devices when applying a train of pulses (5 V, 500 ns) with varying time intervals. To investigate the origin of these temporal properties, pulse measurements at 1 MHz were performed between Pd-Au and Pd-Pd terminals, using the device structure shown in **Fig. S14(a)**. When it comes to high-frequency behavior, capacitances associated with the devices must also be taken into account. For the Pd-Au configuration (**Fig. S14(b)**), there is an H-NNO capacitance between the Pd electrode and the

NNO substrate. Since the Au terminal is grounded and the resistance of the NNO film is significantly lower than that of H-NNO, the NNO substrate can be approximated as ground. Unlike Pd-Au, where the H-NNO capacitance is only between the Pd electrode and the NNO substrate (which acts as a ground), Pd-Pd devices introduce **two H-NNO capacitances** due to the symmetric placement of Pd electrodes on both sides of the NNO layer. In a Pd-Pd configuration, the NNO substrate does not act as a perfect ground. Instead, it develops a floating potential based on the resistance ratio of the two hydrogen clouds. This introduces an additional $LaAlO_3$ substrate capacitance, which influences the high-frequency response (**Fig. S14(c)**).

The real-time measured current response under a pulse train applied to different terminals is shown in **Fig. S15**(a). For the Pd_1-Au and Pd_2-Au configurations, the devices exhibit a fast current response that closely follows the applied voltage pulses with negligible delay. The overall current level at a 5 V bias shows a slight decrease as more pulses are applied, which can be attributed to the expansion of the hydrogen cloud beneath the Pd electrode, resulting in increased resistance when a positive voltage is applied to the Pd electrode. In contrast, the Pd_1-Pd_2 configuration demonstrates a slower current response with an obvious delay. Additionally, the current exhibits accumulation behavior during pulse application and decay behavior after the pulse is removed. This behaviour can be attributed to the introduction of $LaAlO_3$ substrate capacitance, which arises due to the presence of two hydrogen clouds associated with the two Pd electrodes. The observed current increase and decrease correspond to the capacitor's charging and discharging processes. **Fig. S15**(b) presents the Cadence simulation results based on the proposed device circuits previously shown in **Figs. S14**(b) and (c). The simulation results closely match the experimental measurements, confirming the proposed mechanism and validating the circuit model.

Fig. S16(a) presents the experimentally measured capacitance values, C_1 and C_2, under 0.1V voltage bias. The capacitance decreases and exhibits a saturating trend as the applied signal frequency increases. The capacitance values at 1 MHz are used for circuit simulation. For the Pd_1-Pd_2 simulation, the initial R_1 and R_2 values were calculated based on the measured current response of Pd_1-Au and Pd_2-Au under a 5 V spike voltage. By combining the measured C_1 and C_2, and the pulse response in Pd_1-Pd_2, the $LaAlO_3$ capacitance value was extracted through simulation as summarized in **Fig. S16**(b). The final simulation results in **Fig. S16**(b) align closely with the experimental measurements, using directly measured R_1, R_2, C_1 and C_2 values, further validating the reliability of the proposed circuit model.

Supplementary Text 5: Modeling of integration-relaxation dynamics of Pd-Pd devices

To quantitatively describe the transient temporal dynamics of the Pd–Pd device under pulsed excitation, we introduce a fitting model that captures the integration–relaxation behavior observed in **Fig. 2h**. Upon application of a write pulse, the device exhibits a rapid increase in current (integration), whereas after pulse removal the current decays gradually (relaxation). The magnitude of current increase depends on the initial current level prior to pulse application: a lower initial current results in a larger relative increase, leading to the gradually saturated accumulation behavior observed experimentally. Conversely, longer time intervals between successive pulses allow greater current decay, resulting in weaker accumulation. These dynamics can be described using two exponential processes representing fast integration and slow relaxation[10]:

$$I(t) = \begin{cases} I_{th} - (I_{th} - I_0)\exp\left(-\frac{t}{t_a}\right) & if\ spike\ occurs\ at\ time\ t \\ (I_0 - I_\infty)\exp\left(-\frac{t}{t_b}\right) + I_\infty & if\ there\ is\ no\ spike\ at\ time\ t \end{cases} \tag{2}$$

where t_a and t_b represent the time constant of fast increase and slow decay, respectively. I_{th} represents the upper limit of the maximum current value that the H-NNO device could reach.

I_0 is the initial current value and I_∞ is the final convergent current value when there are no more spikes. To further fit this behavior and extract the corresponding values for t_a, t_b, we normalize the measured current by dividing it by I_{th} and discretized the continuous dynamics into discrete time steps, each with a duration of Δt = 500 ns. The derivative form of these two exponential functions could be replaced by:

$$dI(t)/dt = (I(t+\Delta t) - I(t))/\Delta t \tag{3}$$

Then the final equation for normalized current I_n is:

$$I_n(t+\Delta t) = \left(1 - \frac{\Delta t}{t_b}\right) * I_n(t) + \sigma_{spike} * \left(\frac{\Delta t}{t_a}\right) * (1 - I_n(t)) \tag{4}$$

Where σ_{spike} equals 1 when a spike occurs at the time step (t+Δt) and 0 otherwise. Equation (4) is used to fit the experimental data in **Fig. 2h**. The extracted time constant t_b is 6.5 µs, matching well with the result from **Fig. 2g**. The dependence of accumulation behavior on pulse amplitude is shown in **Fig. S17**. Increasing the pulse amplitude enhances the accumulation strength I_{th} while reducing the decay time constant t_b, indicating faster relaxation at higher voltages. This trade-off between response speed and accumulation strength provides a tunable parameter space for optimizing the spatiotemporal processing dynamics.

Supplementary Text 6: Systematic measurements on devices with varying electrode distances and pad sizes

Statistical analyses were performed on Pd–Au devices exhibiting non-volatile switching behavior and Pd–Pd devices exhibiting volatile transient dynamics to evaluate device uniformity and reliability. Measurements were conducted at multiple locations across a 2-inch wafer to assess large-area consistency. The results (**Figs. S18-S25**) show stable resistance switching with low cycle-to-cycle and device-to-device variability, endurance up to 10^6 switching cycles, and reproducible transient responses across 30 devices. These results confirm uniform and reliable device behavior required for array-level spatiotemporal processing and computing.

Supplementary Text 7: Systematic measurements on devices with varying electrode distances and pad sizes

For devices with different electrode distances, the resistance is primarily governed by the hydrogen cloud that forms beneath the Pd electrode rather than the overall channel length. Consequently, electrode spacing has little influence on resistance characteristics, as confirmed by our measurements. As shown in **Fig. S26**, Pd–Au devices with electrode spacings of 10 µm and 160 µm exhibit nearly identical resistance values, indicating negligible dependence on spacing despite the large variation. **Fig. S27** further compares both Pd–Au and Pd–Pd devices with electrode spacings of 5 µm and 10 µm, showing consistent non-volatile switching and transient dynamics across distances. These results demonstrate that electrode spacing does not significantly affect device performance, suggesting that scaling down the electrode distance remains feasible without compromising functionality.

In contrast, the Pd pad size has a pronounced effect. Larger pads reduce resistance but simultaneously increase device capacitance, which scales approximately with the pad area. As a result, the overall RC time constant increases with pad size, directly influencing transient dynamics. As shown in **Fig. S28**, devices with larger pads generally exhibit higher current levels due to their lower resistance, while also reaching saturation more quickly under pulse trains because the slower decay associated with the larger RC allows current to build up more easily. Together, these results indicate that while electrode spacing does not significantly alter device performance, pad size provides an effective tuning knob for both resistance and temporal dynamics.

Supplementary Text 8: Calculation of effective resistance between Pd Node and Nickelate Film

The calculation of the effective resistance between a Pd node surrounded by a hydrogen cloud and the NNO film starts by considering a thin square shell of thickness *dx* as shown in **Fig. S29a**. From the COMSOL simulation in **Fig. S29b**, it is evident that the net current flow is perpendicular to the edges of the Pd node. The resistance *dR* of this shell is given by:

$$dR = \rho * \frac{dx}{Area} = \frac{\rho * dx}{4*(L+2*x)*t} \tag{5}$$

where ρ is the resistivity of the hydrogen cloud, *L* is the length of the Pd node, *t* is the thickness of the nickelate film and x is the distance from the Pd node. To calculate the total resistance R between the Pd node and the NNO film, dR is integrated over the thickness of the hydrogen cloud from x=0 to $x=x_0$:

$$R = \int_{x=0}^{x=x_0} \frac{\rho dx}{4(L+2x)t} \tag{6}$$

Evaluating the integral yields:

$$R = \frac{\rho}{8t} \ln\left(1 + 2\frac{x_0}{L}\right) \tag{7}$$

Supplementary Text 9: Mathematical formulation of substrate-mediated global coupling

To clarify the distinction between substrate-mediated global coupling and conventional nearest-neighbor reservoir computing (RC), we provide here a concise mathematical formulation describing the pairwise coupling resistance between two electrodes in the Pd/H–NNO array.

In this model, *i* and *j* denote the indices of individual electrodes in the Pd/H–NNO array, while *m* represents a summation index running over all *N* electrodes in the array (*m*=1,2,…,*N*). The binary variable u_i(t) represents the input applied to electrode *i* at time step *t*: u_i=1 corresponds to an applied voltage pulse with amplitude V_0, and u_i=0 indicates no applied bias. Each electrode possesses a local conductance $G_i=1/R_i$ that depends on the effective hydrogenated thickness x_i(t), which evolves dynamically according to the local electric field E_i. The expansion/compression coefficient k_i quantifies how x_i changes with the local electric field, and is approximately treated as a constant *k* across all electrodes.

The total conductance with the nodes under applied input voltage V_0 and with grounded nodes are expressed as

$$G_+ = \sum_m u_m G_m \,;\; G_- = \sum_m (1-u_m) G_m \tag{8}$$

So the total conductance is as follows:

$$G = G_+ + G_- = \sum_m G_m \tag{9}$$

Because the substrate behaves as a common resistive node linking the electrodes, its steady potential V_{steady} under a specific pulse configuration can be determined by a simple voltage division between the total conductance of biased (G_+) and grounded (G_-) nodes:

$$V_{steady} = V_0 \frac{G_+}{G_+ + G_-} = V_0 \frac{\sum_m u_m G_m}{\sum_m G_m} = V_0 u_{ave} \tag{10}$$

Where u_{ave} denotes the conductance-weighted average input level across the entire array. This quantity represents the global reference potential of the conductive substrate and forms the mathematical origin of the non-local coupling effect.

At each programming step, the substrate potential $V_{sub}(t)$ evolves according to:

$$V_{sub}(t+\Delta t) = V_{steady} + \left(V_{sub}(t) - V_{steady}\right)e^{-G\frac{\Delta t}{C}} \tag{11}$$

For simplification, we assume that substrate potential V_{sub} rapidly approaches its steady-state value V_{steady} within each pulse duration, so that $V_{sub} = V_{steady}$ holds at every time step. Consequently, the local electric field beneath electrode i is expressed as:

$$E_i(t) = \frac{V_{electrode,i} - V_{sub}}{x_i(t)} = \frac{V_0(u_i(t) - u_{ave}(t))}{x_i(t)} \tag{12}$$

The hydrogenated thickness evolves dynamically according to the local electric field:

$$x_i(t) = x_i(t-1) + kE_i(t-1) = x_i(t-1) + \frac{kV_0}{x_i(t-1)}(u_i(t-1) - u_{ave}(t-1)) \tag{13}$$

The local resistance of each node is given by (detailed explanation is shown in **Supplementary Text 6**):

$$R_i = \frac{\rho \ln\left(1+2\frac{x_i}{L_{electrode}}\right)}{8t_{film}} \tag{14}$$

Where ρ, t_{film} , and $L_{electrode}$ are the resistivity, film thickness, and electrode length, respectively. Linearizing this expression around the previous time step t-1 gives:

$$\Delta R_i \approx \alpha_i(t-1)\left(u_i(t-1) - u_{ave}(t-1)\right),\ \alpha_i \propto \frac{\rho k V_0}{t_{film} x_i(t-1)(L_{electrode} + 2x_i(t-1))}$$

For the pairwise coupling resistance defined as $R_{ij} = R_i + R_j$, the state-update equation becomes:

$$R_{ij}(t) \approx R_{ij}(t-1) + \Delta R_i + \Delta R_j = R_{ij}(t-1) + \alpha_i(t-1)\left(u_i(t-1) - u_{ave}(t-1)\right) + \alpha_j(t-1)\left(u_j(t-1) - u_{ave}(t-1)\right) \tag{15}$$

If the electrodes are geometrically uniform ($x_i(t-1) \approx x_j(t-1)$), this reduces to:

$$R_{ij}(t) \approx R_{ij}(t-1) + \alpha\left(\left(u_i(t-1) - u_{ave}(t-1)\right) + \left(u_j(t-1) - u_{ave}(t-1)\right)\right) \tag{16}$$

In this formulation, the terms u_i and u_j represent local excitation applied to individual electrodes, while u_{ave} captures the global, substrate-mediated potential coupling all nodes through the conductive NNO layer. The additional u_{ave} term is absent in conventional nearest-neighbour RC, where only adjacent nodes interact directly. Here, because V_{sub} is determined collectively by all nodes across the wafer, each node's state evolution depends on the entire network. This substrate-mediated averaging results in a global coupling resistance that is effectively distance-independent across the array.

Supplementary Text 10: Linear output layer based on Pd-Au devices

The linear output layer array design is based on Pd-Au devices. The Au terminal serves as a bit line to collect the current from the surrounding Pd pads (word lines). The generated current from each Pd pad depends on its individual hydrogen cloud resistance and the applied input voltage level. Unlike the spatiotemporal processing layer design, where coupling effects are integral, the multiplication of weights and linear weighted sum operations require isolation between each Pd electrode. A common Au line functions to collect the current from all Pd pads and linearly sum them according to Kirchhoff's Law. To achieve this, we designed a surrounding Au ring around each Pd pad to ensure isolation between neighboring Pd electrodes (**Fig. S30a**).

The initial resistance distribution map of the 14×10 Pd-Au array was obtained by applying a 0.1 V voltage bias to the target Pd electrode, while floating all other Pd electrodes and grounding the Au electrode (**Fig. S30b**). The measured resistance values ranged from 80 to 200 kΩ across the 140 Pd terminals, demonstrating good uniformity. To validate the design, we

measured the output current from the Au pad by configuring the surrounding Pd electrodes with various voltage inputs (**Fig. S30c**). The expected output current was calculated by linearly summing the contributions from each Pd pad using the measured resistance map. The close agreement between the measured and expected values confirms the linear summation behavior of the Pd-Au device array, extending across varying voltage biases. This demonstrates the array's consistent and reliable performance under different operating conditions.

Supplementary Text 11: AudioMNIST task and spatiotemporal processing configuration
The AudioMNIST dataset consists of spoken digits (0–9) recorded from six speakers. In this work, the first 3,000 samples (approximately 10% of the dataset) were used for training and evaluation. Audio signals were preprocessed using the Lyon's ear model to emulate cochlear frequency analysis. The resulting signals were bandpass filtered into 64 frequency channels with varying amplitudes and subsequently sampled into 50 time steps. Each sample was normalized and converted into binary spike trains using a fixed threshold. The 64 spike trains were encoded as voltage pulse sequences and applied to the Pd electrodes of the spatiotemporal processing layer. The resulting current responses were used as input features for a Pd–Au linear output layer, where classification was performed using linear regression.
To isolate the role of spatiotemporal dynamics arising from protonic coupling, two processing configurations were considered: a temporal-only case and a spatiotemporal case. In the temporal-only configuration, each input spike train was assigned to an isolated Pd–Pd junction, such that individual devices operated independently without spatial coupling and exhibited only nonlinear temporal decay. In the spatiotemporal configuration, each spike train was randomly assigned to two Pd pads, enabling spatial interactions between neighboring nodes via protonic coupling in addition to intrinsic temporal dynamics. For both configurations, 128 Pd pads were used with identical initial hydrogen cloud thickness distributions ranging from 2 to 3 μm. All other parameters were kept identical between the two cases.

Supplementary Text 12: Mahalanobis distance calculation
To demonstrate the advantages of the dynamic characteristics of our spatiotemporal processing layer design, we calculate the average Mahalanobis distance between pairs of digit classes using the output generated in three cases: no processing, temporal only and spatiotemporal (**Fig. S33**). The Mahalanobis distance D(x,y) between two classes, with mean feature vectors x and y, is defined as:

$$D(x,y) = \sqrt{(x-y)^T \Sigma^{-1} (x-y)} \tag{17}$$

where Σ is the covariance matrix of the features.
For the no-processing case, feature vectors were obtained by linearly summing the accumulated spike counts up to the sampling time step. In contrast, for the temporal-only and spatiotemporal cases, the output current from each Pd terminal was used. As shown in **Fig. S33**, the results show that increasing the number of sampled time steps enhances class separation across all three cases. More importantly, the spatiotemporal processing layer consistently exhibits the highest pairwise distance compared to the temporal-only and no-processing cases. This suggests that incorporating spatial interactions improves the ability to separate digit classes, thereby enhancing feature transformation and improving classification performance.

Supplementary Text 13: Energy comparison of different processing systems
Table S1 presents a comparative analysis of energy consumption across various hardware processing systems, including conventional CPU and FPGA implementations, oxide-based memristor, nanowire networks, electrochemical transistors, and our proposed H-NNO system. The energy per input metric quantifies the energy consumed per input voltage pulse during processing operations, derived from the applied pulse configuration and the resulting current

levels in each system. In our case, this is estimated as: E = 5V × 80μA × 500ns = 0.2nJ (refer to **Fig. 2g** in the main text). For the average energy per spoken digit, we performed calculations based on the same 3000 spoken digit samples used throughout our task simulation and report the average value. In our H-NNO spatiotemporal processing layer, 128 Pd nodes are used. Each Pd node receives a voltage spike train V(t) and generates output current I(t) based on the collective voltage dynamics of all nodes. The energy per digit is obtained by integrating the instantaneous power over time for each node and summing across all 128 nodes:

$$E_{spoken\ digit} = \sum_{i=1}^{128} \int V_i(t)I(t)dt \quad (18)$$

For temporal-only cases in previous works, where each node operates independently without spatial interactions, we calculated the energy per spoken digit using the same 3000 samples by calculating the average number of pulses N in each spoken digit which is 157 pulses in our case and applying: $E_{spoken\ digit}$=N×Energy/Input. This ensures a consistent comparison across different implementations. As can be seen, the energy consumption per spoken digit in our case is lower than all the temporal only works using CPU, FPGA and memristors, despite our processing system incorporating both node interactions and nonlinear temporal characteristics. For spatiotemporal processing in other references with spatial interaction across the whole system, system-level spoken digit simulations were not performed, making it difficult to estimate their total energy per spoken digit. However, by comparing their energy per input, we observe that other spatiotemporal processing hardware require millisecond-scale operation and consume orders of magnitude higher energy per input than our H-NNO spatiotemporal processing layer, which operates at a nanosecond time scale (500 ns) with just 0.2 nJ per input.

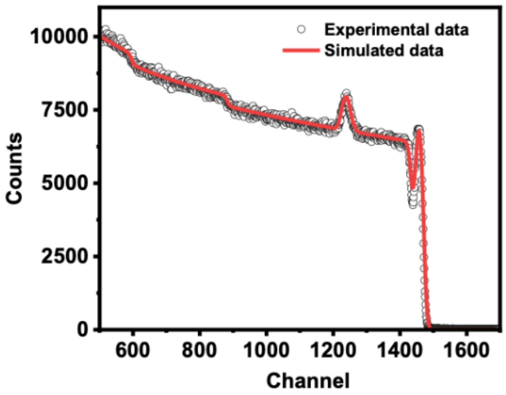

Supplementary Fig. 1: Rutherford backscattering spectroscopy (RBS) data for a representative pristine $NdNiO_3$ (NNO) thin film on $LaAlO_3$ (LAO) substrate. Here, a 2.3 MeV He^{2+} ion beam of 2 mm diameter bombards the thin film, with backscattered particles observed. The x-axis (channels) indicates the energy of the backscattered particles, where higher channel numbers correspond to heavier particle. Simulated plot (in red) using SIMNRA software can be utilized to estimate the atomic density and calculated to be 81.0×10^{15} atoms/cm^2 for Nd, 66.6×10^{15} atoms/cm^2 for Ni, and 212.4×10^{15} atoms/cm^2 for O. The thickness of the NNO film is estimated to be ~50 nm.

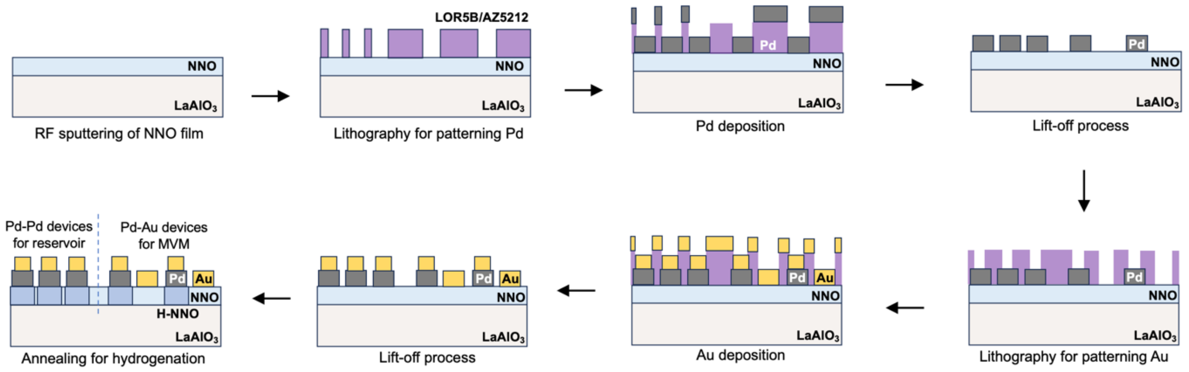

Supplementary Fig. 2: Schematic of the fabrication process flow for Pd-Pd and Pd-Au nickelate devices.

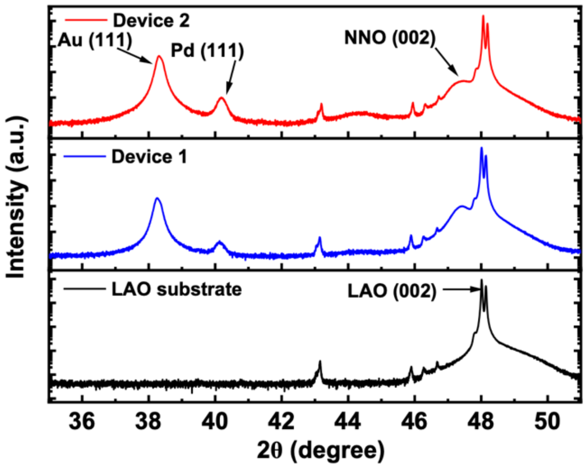

Supplementary Fig. 3: X-ray diffraction (XRD) data for LAO substrate, H-NNO/LAO films at Pd-Au junction (device 1) and Pd-Pd junction (device 2). XRD of bare LAO substrate shows characteristic (002) peak at ~47.94°. XRD of device 1 and device 2 show the presence of Au and Pd electrodes as Au (111) and Pd (111) peaks at 38.4° and 40°, respectively. The (002) peak of hydrogenated NNO is observed at ~47.70°, consistent with literature[9].

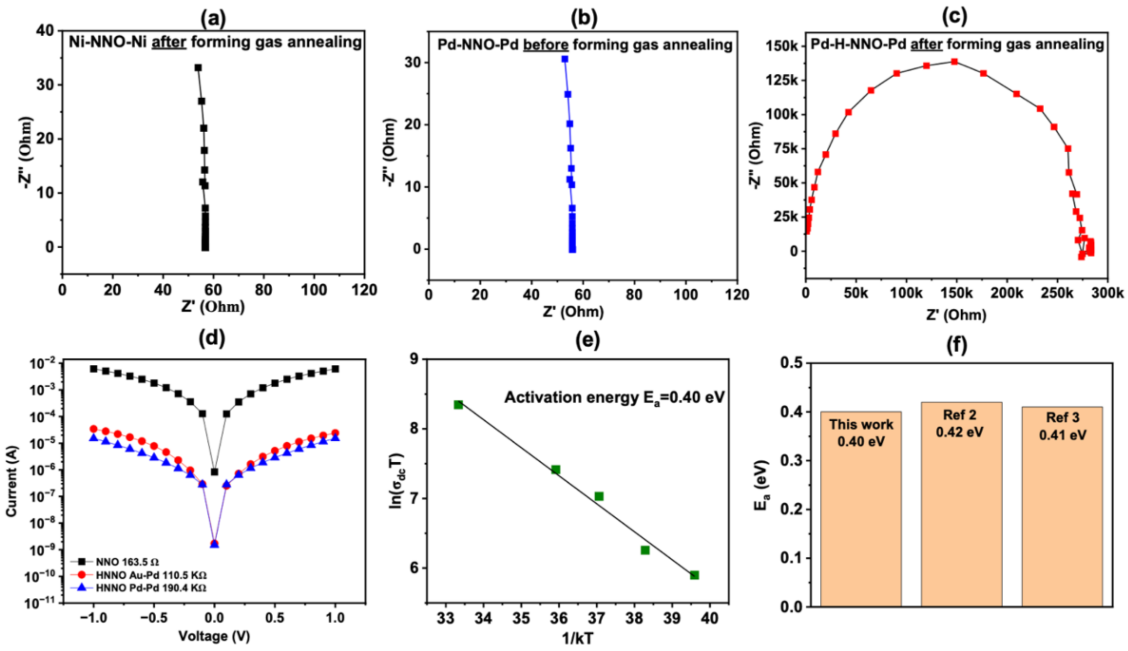

Supplementary Fig. 4: Room temperature Impedance spectroscopy study of NNO to H-NNO phase transition. **(a)** Nyquist plots of a control NNO device with non-catalytic Ni electrodes, the film remains highly electrically conducting even after forming gas annealing. Nyquist plots of a NNO device with catalytic Pd electrodes **(b)** before and **(c)** after forming gas annealing shows distinct changes in the nature of the plots from linear to semicircular and indicates hydrogenation induced phase transition to an insulating state (NNO to H-NNO). **(d)** I–V characteristics in log scale before and after forming-gas annealing, showing resistance increase from 163.5 Ω (undoped) to 110.5 kΩ (Au/H-NNO/Pd) and 190.4 kΩ (Pd/H-NNO/Pd). **(e)** Arrhenius plot from temperature-dependent *dc* conductivity gives the activation energy (E_a) of proton migration in H-NNO with an activation energy of 0.40 eV (from the slope). **(f)** The activation energy (E_a) derived from EIS of our samples compared with literature (both experimentally[2] and theoretically[3]).

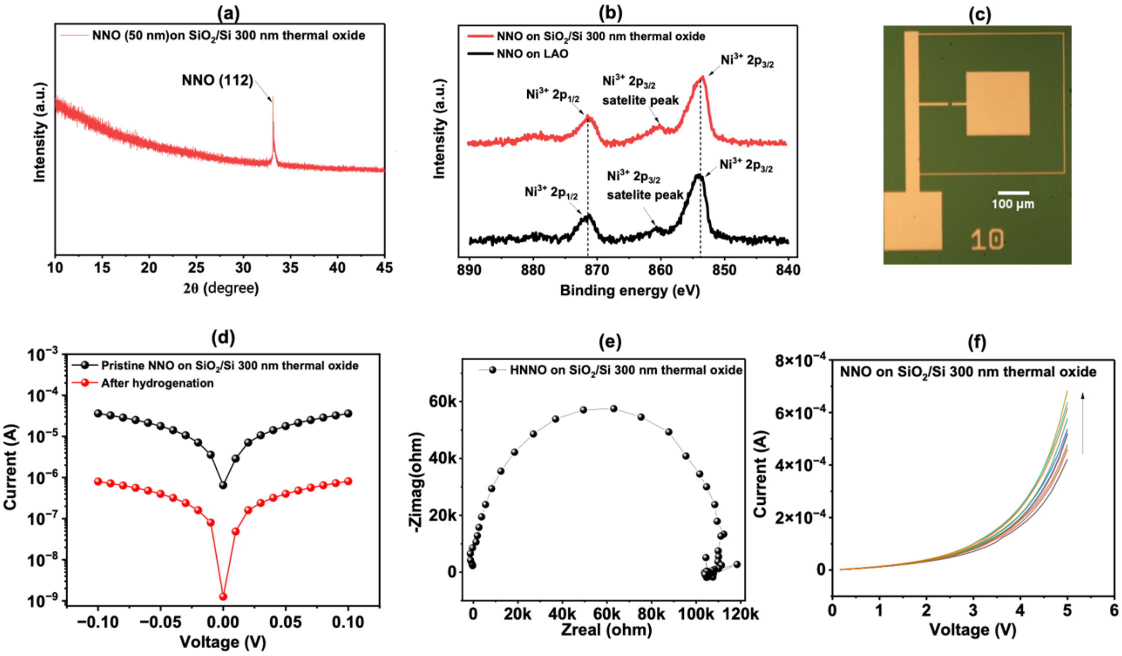

Supplementary Fig. 5: **(a)** XRD of NNO film grown on 300 nm thermal oxide SiO_2/Si substrate showing characteristic (112) film peak of NNO. **(b)** Ni 2p core-level XPS spectra of NNO grown on SiO_2/Si substrate, and comparison to NNO grown on LAO substrate, both showing consistent binding energies confirming the expected Ni oxidation state. **(c)** Representative optical image of a fabricated NNO on SiO_2/Si substrate with 10 µm gap. **(d)** I-V data shows significant change in resistance before and after hydrogen doping by forming gas annealing. **(e)** Nyquist plot from EIS measurement for HNNO on SiO_2/Si substrate shows insulating characteristics as expected from doping. **(f)** Consecutive I-V sweeps show tunable resistance states, arising from proton drift.

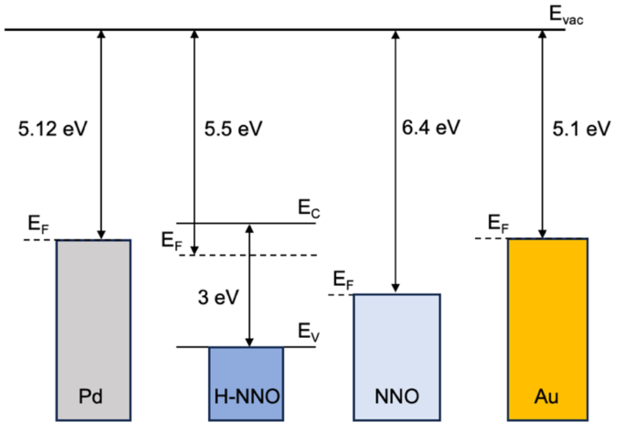

Supplementary Fig. 6: The energy band diagrams of Pd, H-NNO, NNO and Au before contacting.

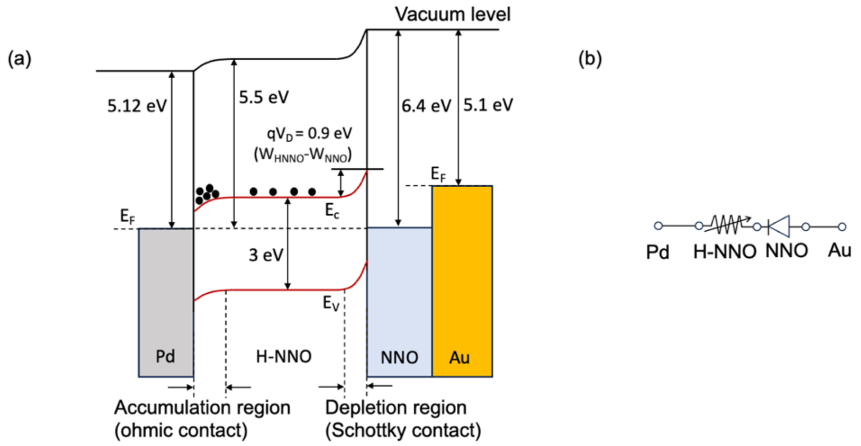

Supplementary Fig. 7: (a) The energy band diagrams of the Pd-Au device after contacting at zero voltage bias. **(b)** The simplified connecting schematic between each layer.

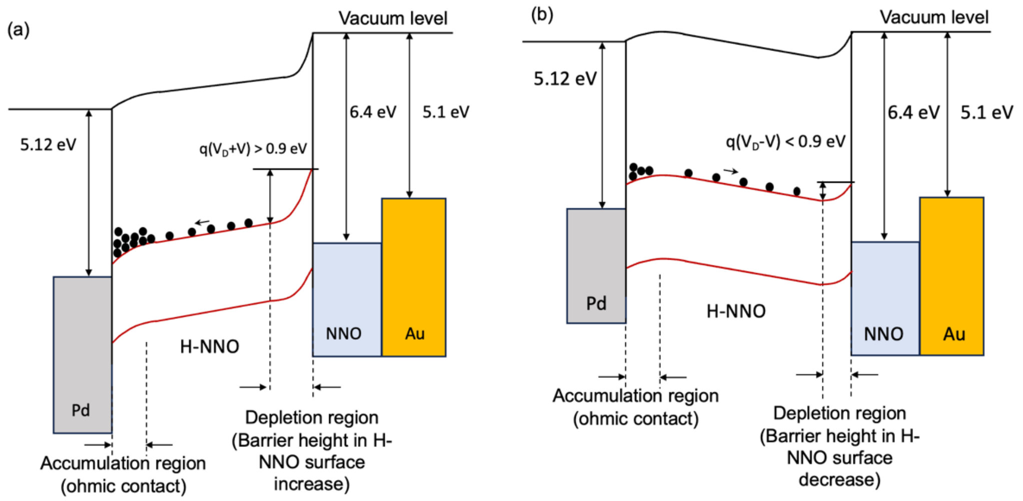

Supplementary Fig. 8: The energy band diagrams of the Pd-Au device under **(a)** positive and **(b)** negative voltage bias.

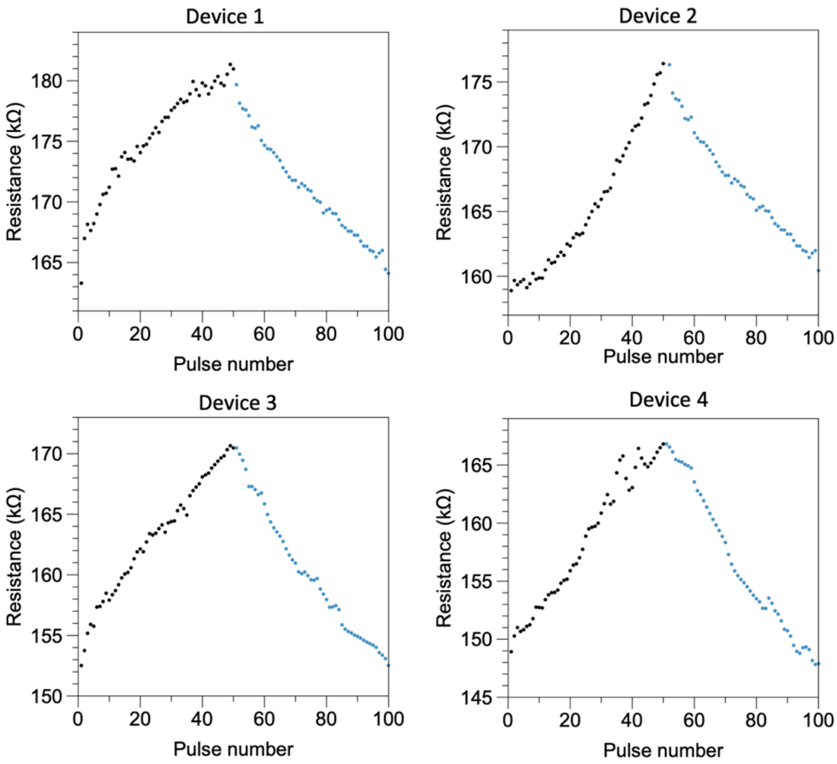

Supplementary Fig. 9: Device resistance update behaviour under a series of positive and negative voltage pulses, showing consistent switching characteristics across different devices within a similar resistance range.

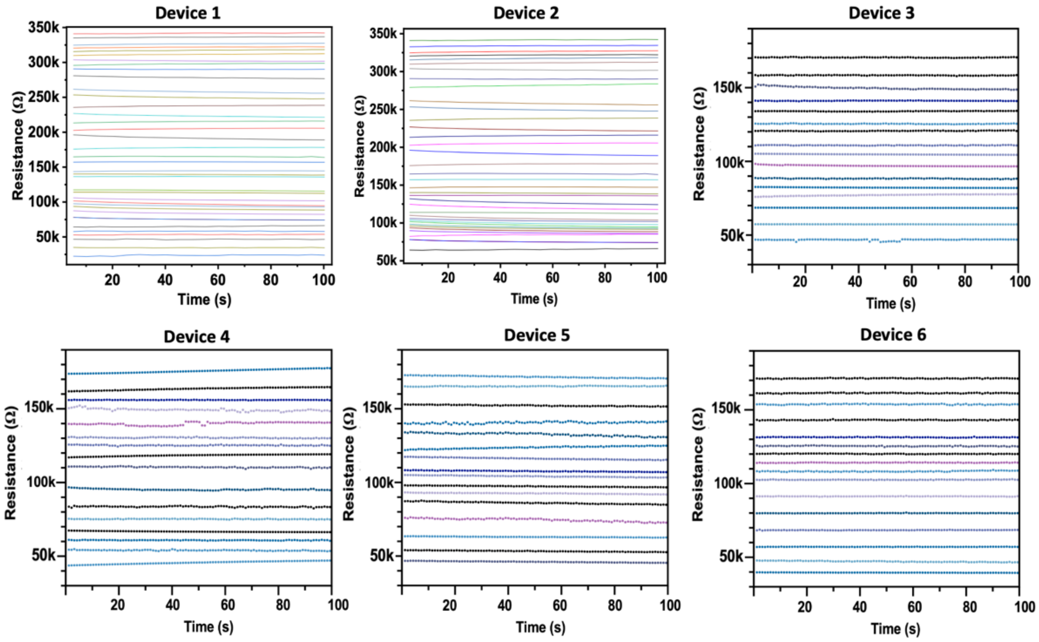

Supplementary Fig. 10: 36 or 16 distinct resistance states across multiple devices within the same resistance range (~40 kΩ to ~350 kΩ for 36 resistance states and ~40 kΩ to ~180 kΩ for 16 resistance states), each retained for 100 s.

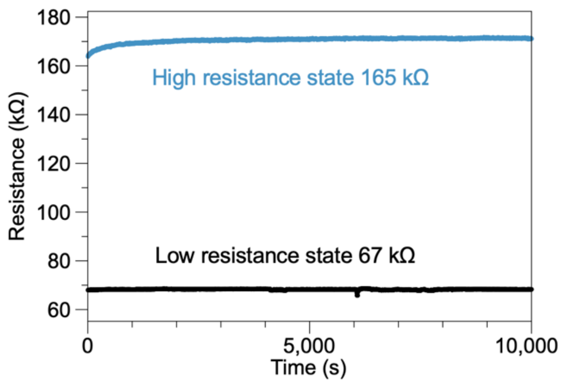

Supplementary Fig. 11: Retention of representative high and low resistance states, demonstrating long-term stability up to 10000 s.

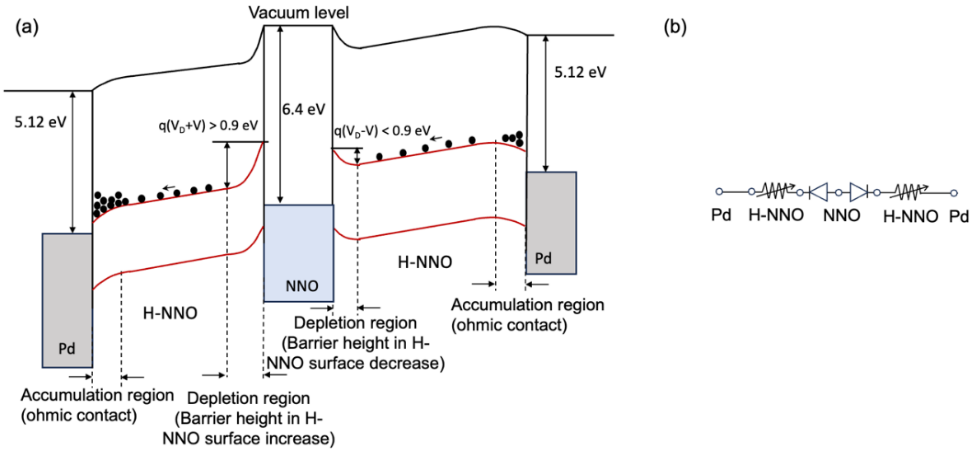

Supplementary Fig. 12: (a) The energy band diagrams of the Pd-Pd device with voltage bias. **(b)** The corresponding simplified schematic.

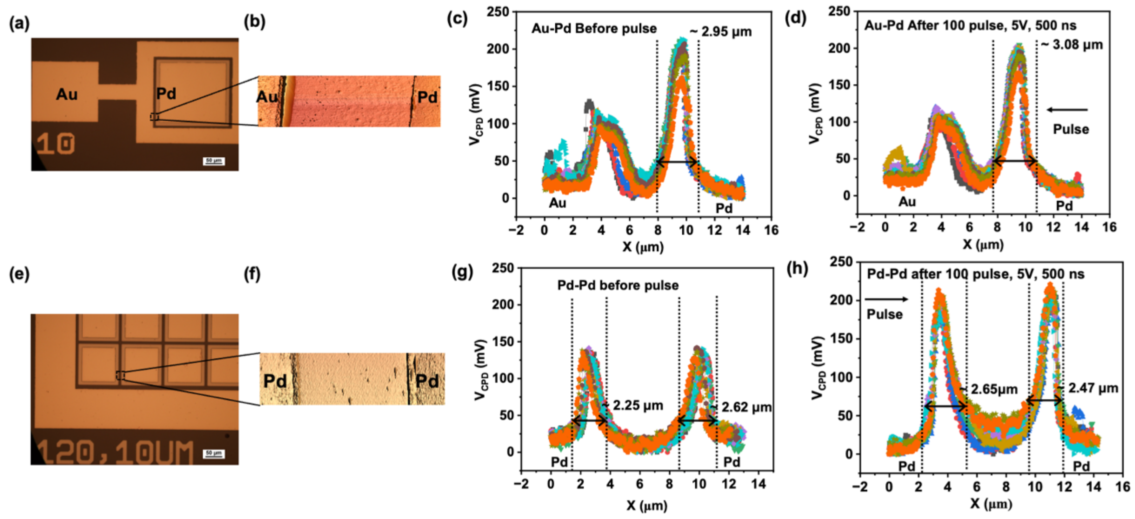

Supplementary Fig. 13: Optical microscope images of fabricated **(a)** Pd-Au and **(e)** Pd-Pd devices with 10 µm channel lengths. Atomic force microscopy (AFM) topography of one **(b)** Pd-Au and one **(f)** Pd-Pd device. Kelvin Probe Force Microscopy (KPFM) line profiles showing contact potential difference (V_{CPD}) before electrical stimulus for **(c)** Pd-Au and **(g)** Pd-Pd devices, with 10 representative line profiles per device. Changes in V_{CPD} line profiles after the application of 100 pulses of 5V with 500 ns pulse width for **(d)** Pd-Au and **(h)** Pd-Pd devices, highlighting the impact of electrical stimuli on the device surface potential.

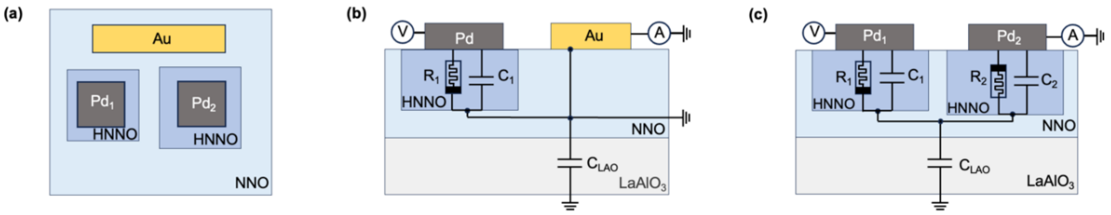

Supplementary Fig. 14: The schematic of **(a)** top view and side views of **(b)** Pd-Au and **(c)** Pd-Pd device structures.

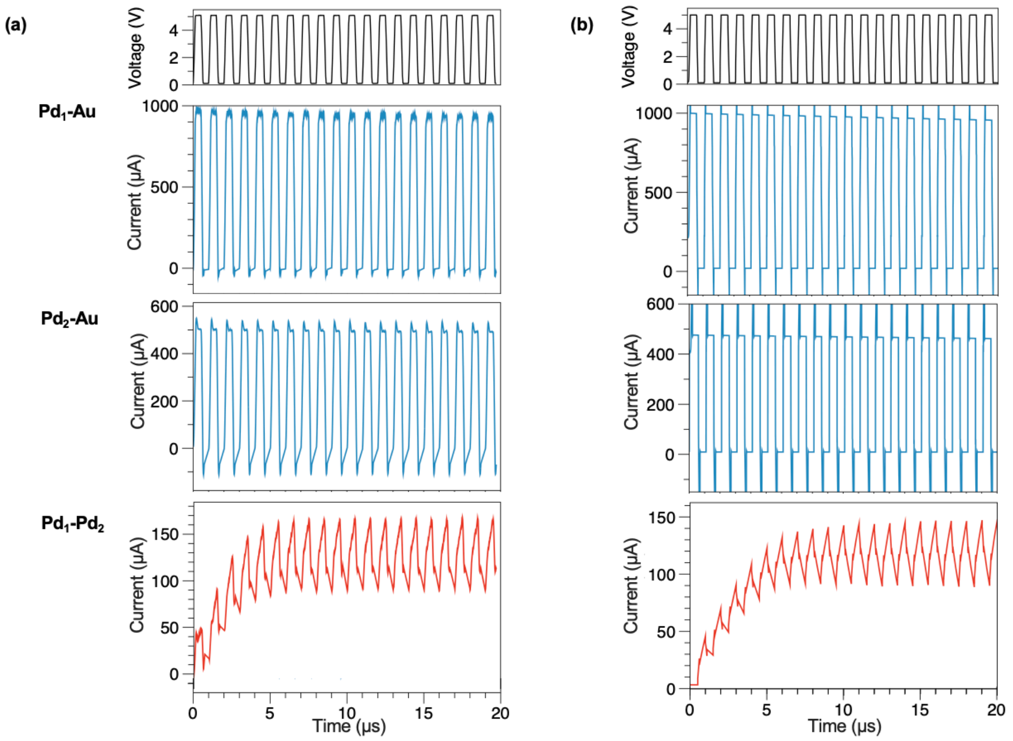

Supplementary Fig. 15: Real-time monitoring of current changes for Pd_1-Au, Pd_2-Au, and Pd_1-Pd_2 configurations under a pulse train (5 V, 500 ns) at 1 MHz. The experimentally measured results **(a)** and circuit simulation results using Cadence **(b)** are consistent.

(a)

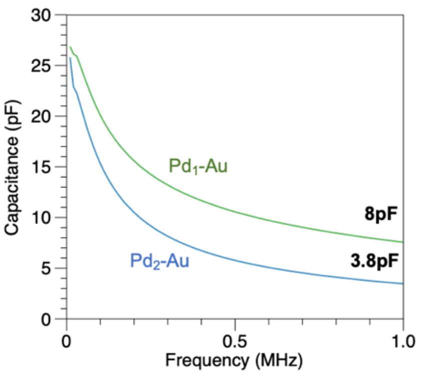

(b)

Circuit parameter	Value
R_1@5V	5 k
R_2@5V	10.5 k
C_1 (parasitic)	8 pF
C_2 (parasitic)	3.8 pF
C_{LAO}	250 pF

Supplementary Fig. 16: (a) The measured capacitance versus frequency between Pd_1-Au and Pd_2-Au. **(b)** The circuit parameter used for Cadence circuit simulation.

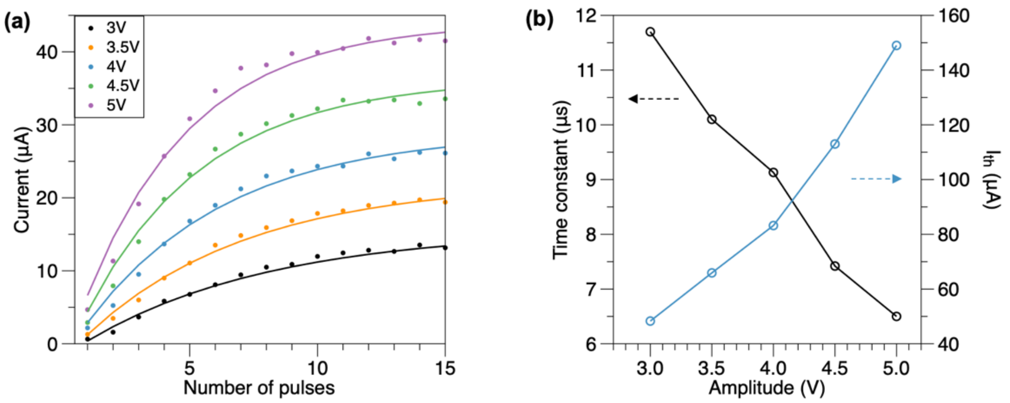

Supplementary Fig. 17: **(a)** Current response under different voltage pulse amplitudes from 3V to 5V. Dots indicate experimentally measured data, and solid lines represent their corresponding fitting results based on Equation 3. Higher pulse amplitudes lead to a greater increase in the output current. **(b)** The extracted device decay time constant and saturated current as functions of applied voltage pulse amplitude. The decay time constant decreases with increasing voltage amplitude, indicating that higher voltages accelerate the relaxation process of hydrogen ions, leading to a faster return to the initial resistance state in volatile devices. Simultaneously, the saturated current value increases with higher voltage amplitude, suggesting enhanced hydrogen migration, which lowers resistance and raises current.

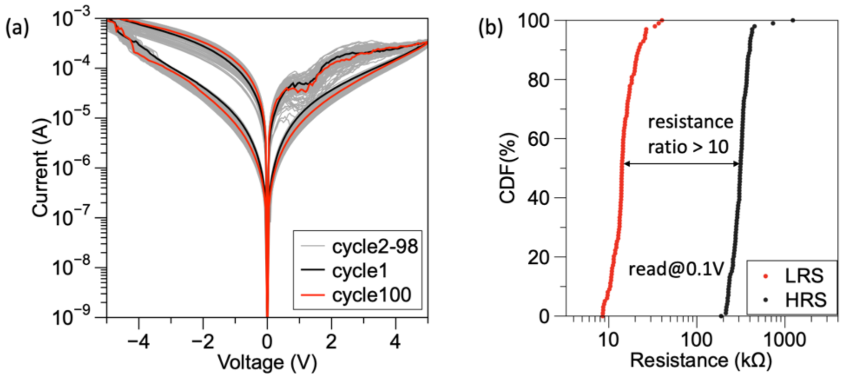

Supplementary Fig. 18: **(a)** DC sweep I–V curves ranging from -5V to 5V for a representative long-term Pd–Au device over 100 consecutive cycles, demonstrating stable switching characteristics. **(b)** Cumulative distribution functions (CDFs) of low-resistance state (LRS) and high-resistance state (HRS) values extracted from the 100 cycles, illustrating the distribution of cycle-to-cycle (C2C) variability.

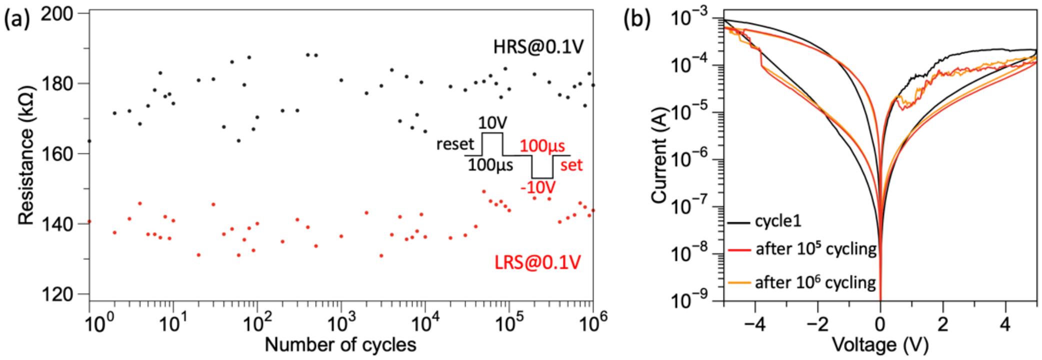

Supplementary Fig. 19: **(a)** Endurance characteristics of a long-term Pd–Au device with successfully SET and RESET over 10^6 pulse cycling operation. **(b)** DC sweeps performed after 10^5 and 10^6 endurance cycles, showing negligible drift compared with the initial cycle, confirming stable long-term C2C performance.

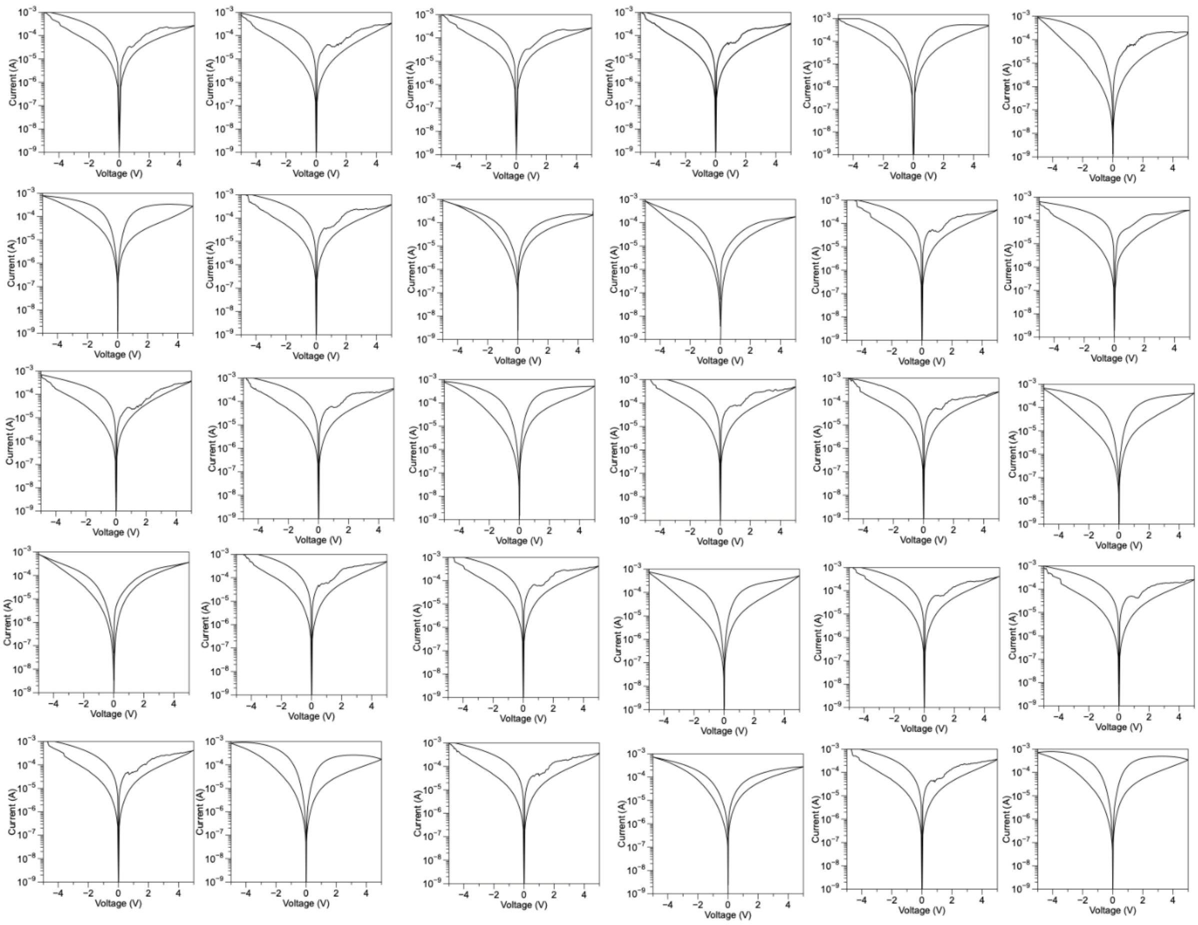

Supplementary Fig. 20: DC sweep I–V curves for 30 long-term Pd–Au devices.

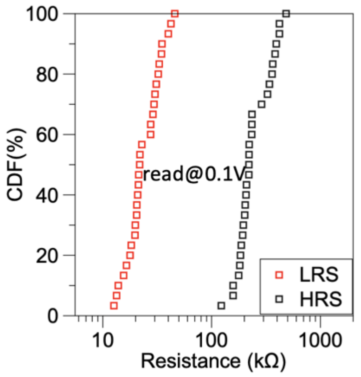

Supplementary Fig. 21: Cumulative distributions functions (CDFs) of LRS and HRS values when read at 0.1V extracted from 30 long-term Pd-Au devices.

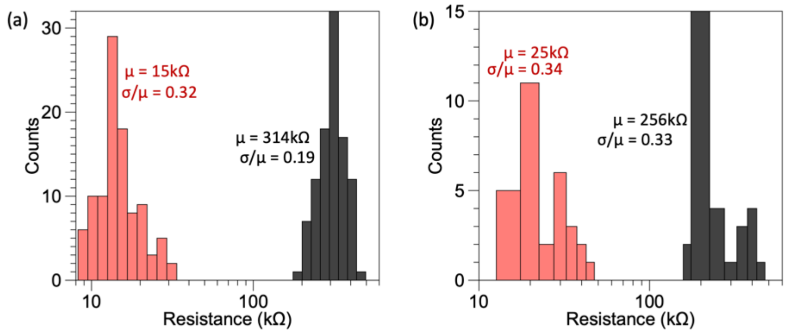

Supplementary Fig. 22: Histograms of LRS and HRS values showing **(a)** cycle-to-cycle (C2C) and **(b)** device-to-device (D2D) variability. The mean values (μ) and relative standard deviations (σ/μ) for each distribution are indicated.

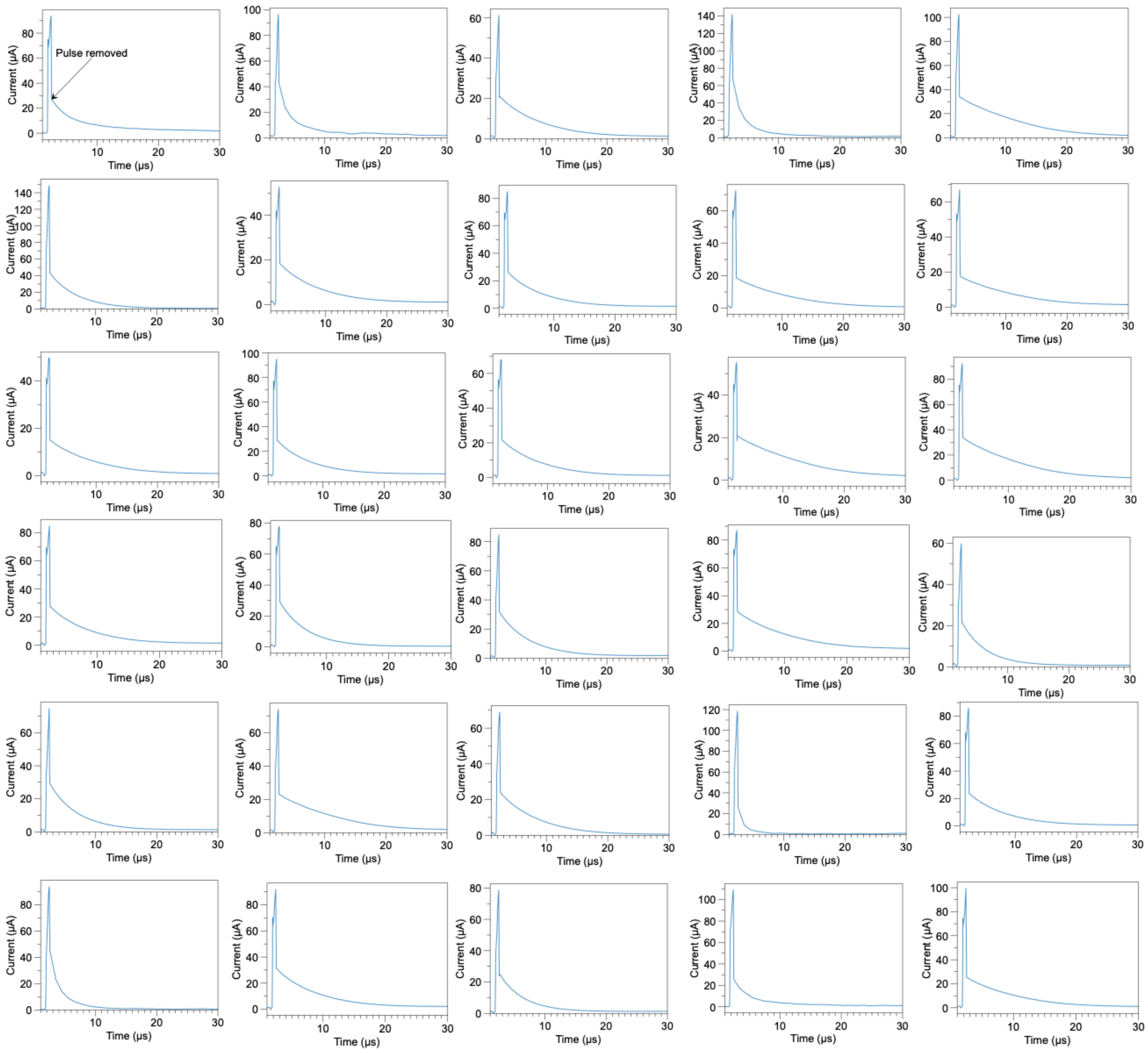

Supplementary Fig. 23: Real-time current decay curves in short-term Pd–Pd devices following a single 5 V, 500 ns pulse, measured under a constant 0.1 V read bias.

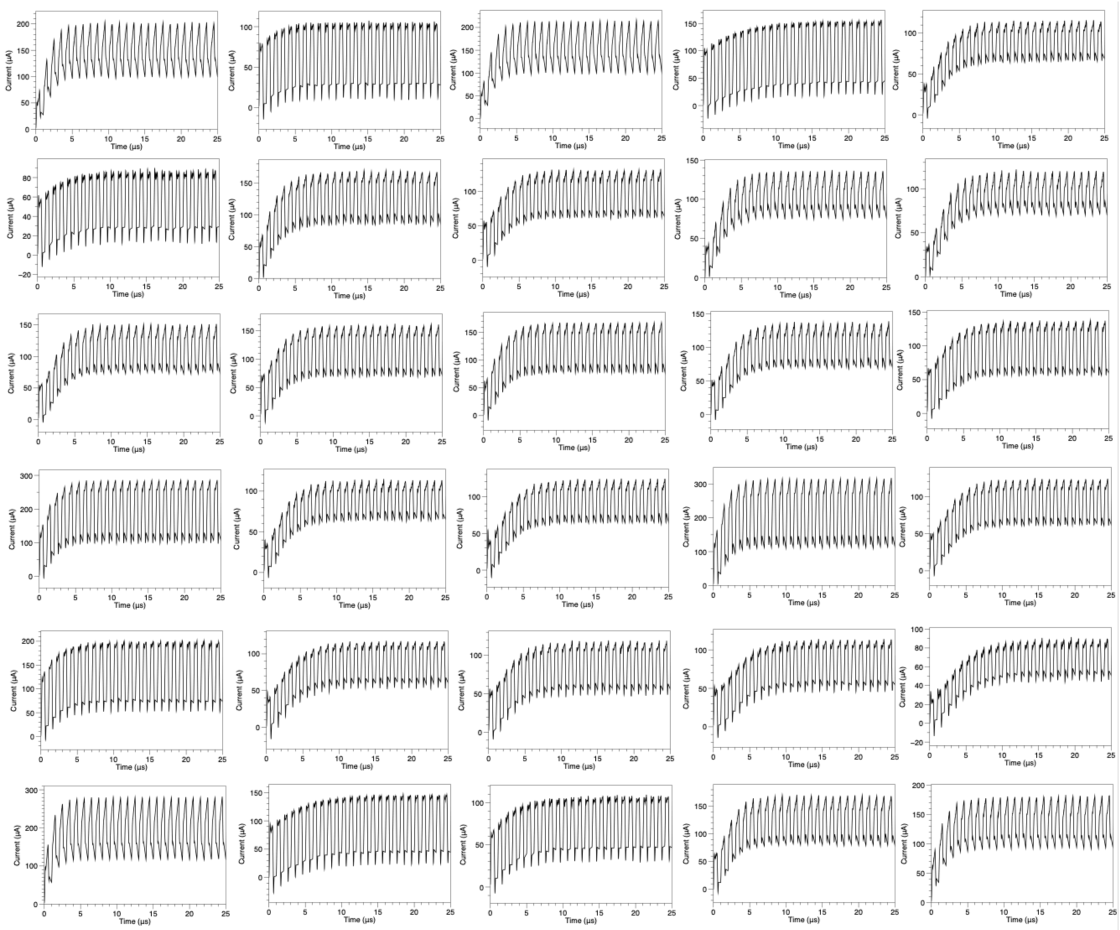

Supplementary Fig. 24: Real-time current responses of short-term Pd–Pd devices under a train of 5 V, 500 ns pulses with 500 ns intervals, showing the approach to steady-state saturation current.

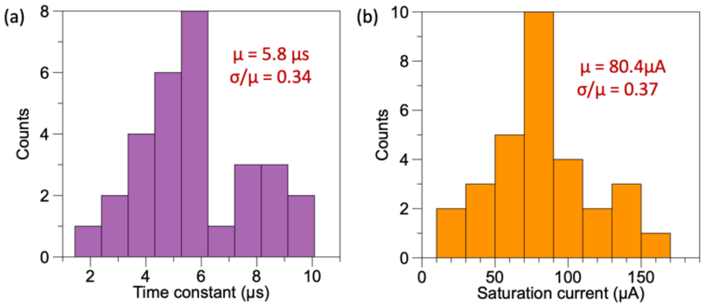

Supplementary Fig. 25: Histograms of **(a)** saturation current and **(b)** decay time constants extracted from 30 short-term Pd–Pd devices. Both distributions exhibit low relative standard deviations (σ/μ), confirming reliable short-term memory (STM) operation.

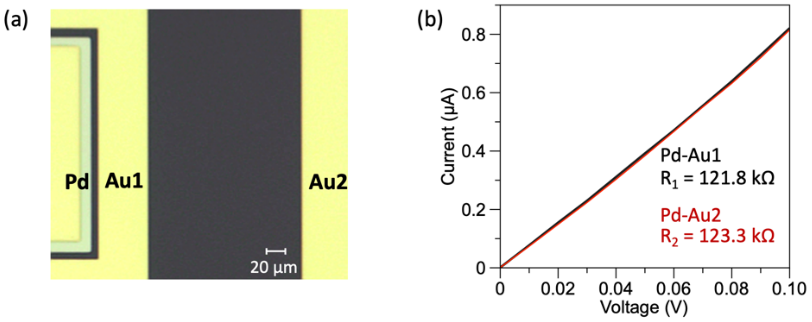

Supplementary Fig. 26: **(a)** Optical microscope image of one Pd pad and two Au pads, forming Pd–Au electrode spacings of 10 µm and 160 µm. **(b)** Corresponding I–V read curves of H-NNO devices with these two spacings. The resistance values show negligible variation despite the large difference in electrode distance.

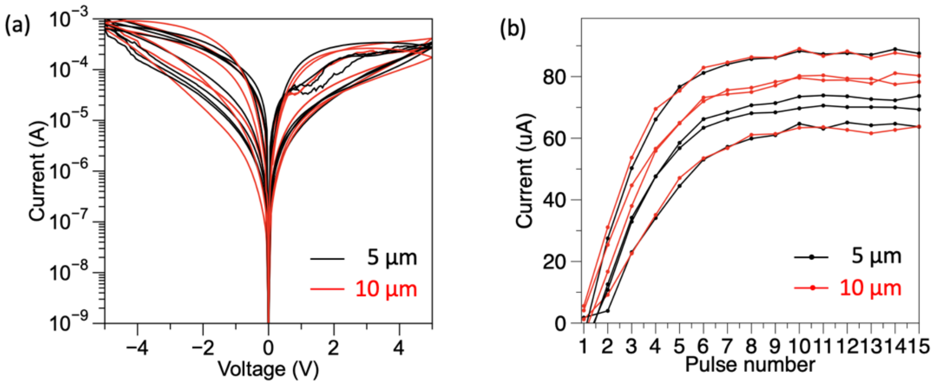

Supplementary Fig. 27: **(a)** DC sweep characteristics of Pd–Au devices with electrode spacings of 5 µm (four devices) and 10 µm (four devices). **(b)** Gradual current increase in Pd–Pd devices with electrode spacings of 5 µm (four devices) and 10 µm (four devices) under a train of voltage pulses (5 V, 500 ns width, 500 ns interval).

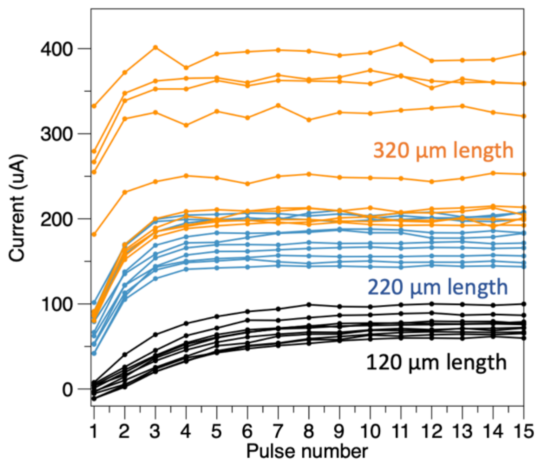

Supplementary Fig. 28: Gradual current increase in Pd–Pd devices with different Pd pad sizes (lengths of 120 µm, 220 µm, and 320 µm) under a train of voltage pulses (5 V, 500 ns width, 500 ns interval).

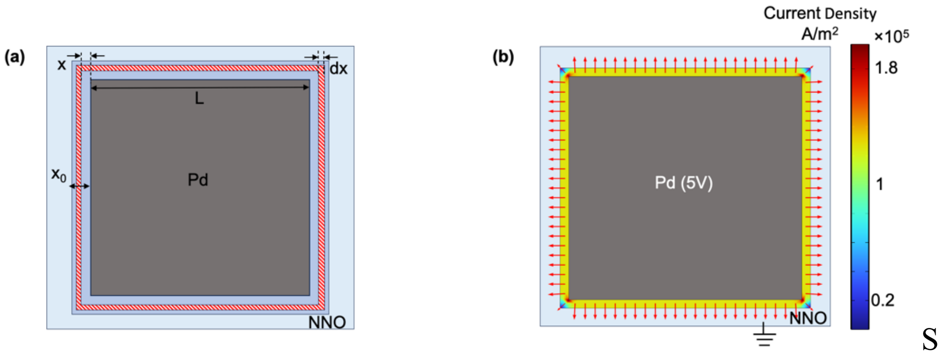

Supplementary Fig. 29: (a) Schematic showing the thin shell of thickness dx and resistance dR, where x denotes the distance from the Pd edge and x_0 is the total hydrogen cloud thickness. Integration of resistance over the cloud thickness yields the total resistance between the Pd node and the NNO film. **(b)** COMSOL simulation of the current distribution in the hydrogen cloud when the Pd pad is biased at 5V and the NNO film is grounded.

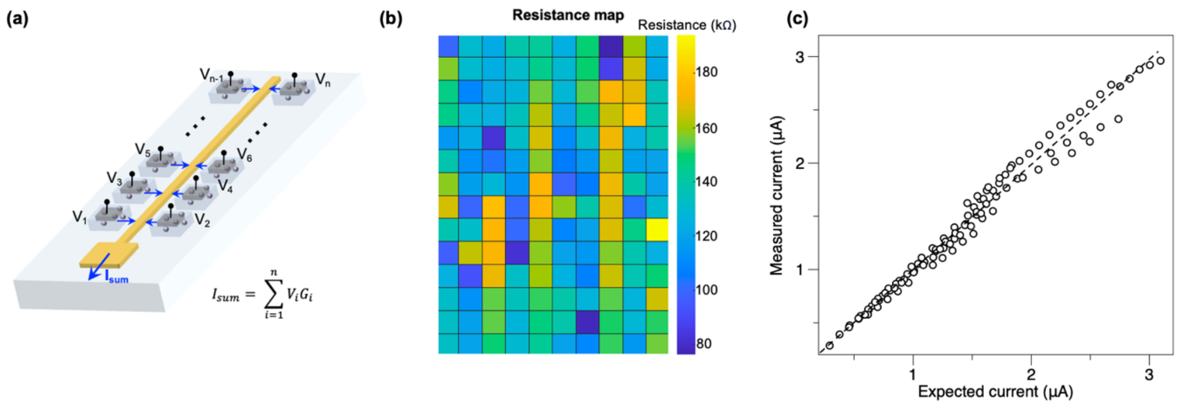

Supplementary Fig. 30: (a) Schematic illustration of linear output layer implemented using Pd-Au devices, with n inputs and single column output. The total output current I_{sum} is expected to be the linear sum of the individual output current from each device. (**b**) Initial resistance distribution of a 14×10 Pd-Au array with resistance variation less than threefold. (**c**) Measured versus expected current outputs I_{sum} based on different input voltage biases ranging from 0.02 V to 0.1 V. Each point represents the total current output for a different input vector composed of varying voltage combinations. The alignment between measurement and expectation suggests that the current outputs in the Pd-Au array add up linearly as expected.

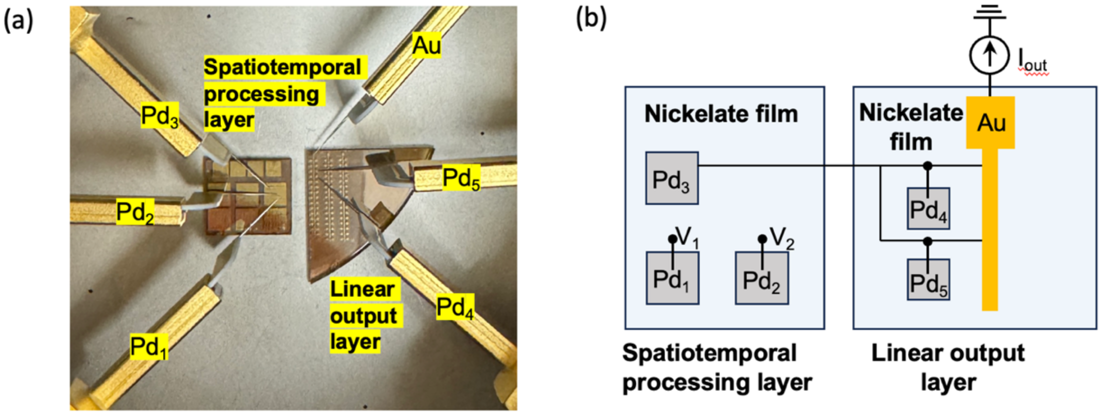

Supplementary Fig. 31: **(a)** Photograph of the experimental setup on a probe station, showing simultaneous measurement of the spatiotemporal processing layer and the linear output layer. The probe tips contact the designated Pd and Au pads, enabling application of voltage pulses and monitoring of output currents. **(b)** Schematic of the measurement configuration. Voltage pulse trains are applied to Pd_1 and Pd_2 via the voltage source terminal, while the Au pad is grounded and its current monitored through the current source terminal. The spatiotemporal processing layer and the linear output layer are interconnected by assigning Pd_3, Pd_4, and Pd_5 pads to the same terminal.

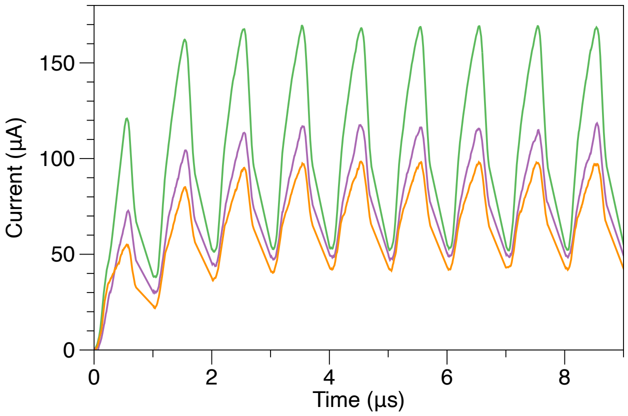

Supplementary Fig. 32: Real-time output current recorded from the Au pad in the linear output layer under voltage pulse trains (5 V amplitude, 500 ns width, 500 ns interval). Different output curves correspond to probing different Pd pads (Pd_4 and Pd_5 can be chosen among 32 pads connected to first Au line)with distinct hydrogen cloud distributions in the linear output layer.

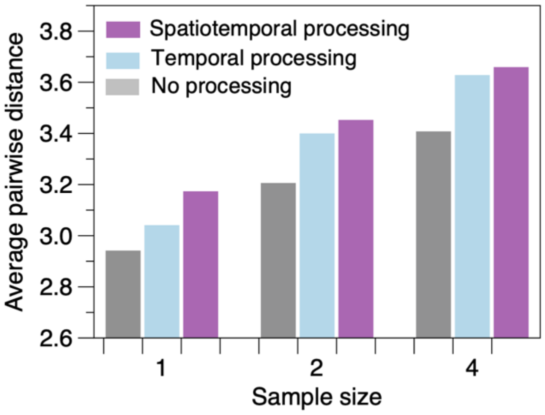

Supplementary Fig. 33: Average pairwise distance between spoken digit classes under three different conditions: no processing, temporal-only processing, and spatiotemporal processing. The x-axis represents the sample size, indicating how many times the entire signal is sampled (once, twice, or four times), while the y-axis shows the average pairwise distance.

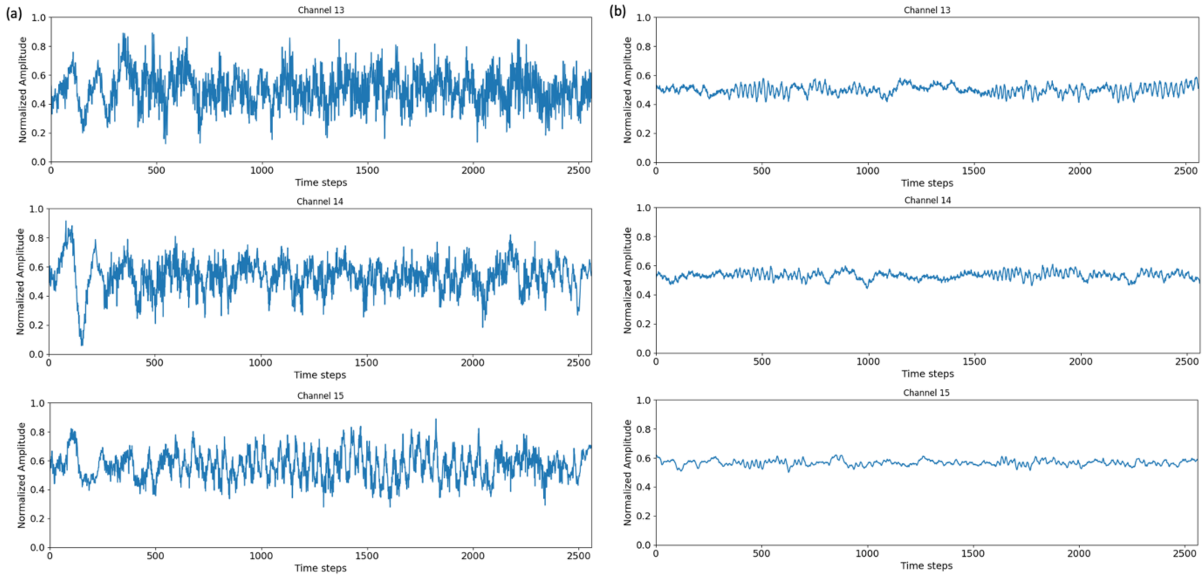

Supplementary Fig. 34: 10-second EEG clips of seizure **(a)** and non-seizure **(b)** signals from channels 13 to 15 in a single patient.

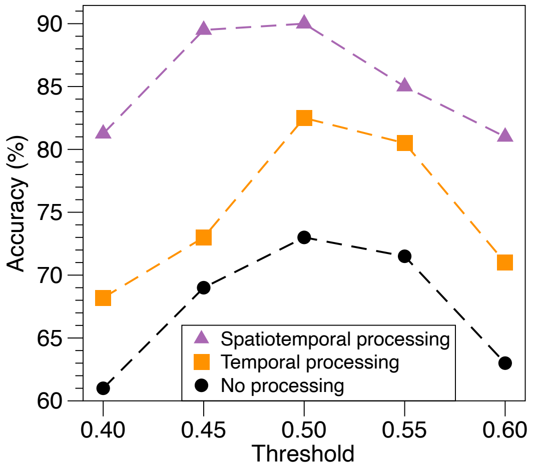

Supplementary Fig. 35: Classification accuracy of EEG signals under three conditions: no processing, temporal-only processing, and spatiotemporal processing (with varying thresholds).

Supplementary Table 1. Estimated energy consumption

Hardware type	Processing mode	Time scale	Energy/Input	Average energy /spoken digit
Nanowire[11]	Spatiotemporal	10 ms	750 μJ	N/A
Electrochemical transistors[12]	Spatiotemporal	100 ms	20 nJ	N/A
CPU[13]	Temporal only	4 μs	54.8 μJ	8.6 mJ
FPGA[13]	Temporal only	1.7 μs	143 nJ	22.5 μJ
WO_x memristor[13]	Temporal only	10 μs	3 nJ	471 nJ
TiO_x/TaO_y memristor[10]	Temporal only	1 ms	120 nJ	18.8 μJ
TiO_x memristor[14]	Temporal only	0.2 ms	10 nJ	1.6 μJ
H-NNO (this work)	Spatiotemporal	500 ns	0.2 nJ	280 nJ

Supplementary References

1 Ko, C. & Ramanathan, S. Stability of electrical switching properties in vanadium dioxide thin films under multiple thermal cycles across the phase transition boundary. *Journal of Applied Physics* **104**, 086105, doi:10.1063/1.3000664 (2008).

2 Taniguchi, Y., Li, H.-B., Hattori, A. N. & Tanaka, H. Comprehensive determination of proton diffusion in protonated NdNiO3 thin film by a combination of electrochemical impedance spectroscopy and optical observation. *Applied Physics Express* **16**, 035501, doi:10.35848/1882-0786/acc004 (2023).

3 Yoo, P. & Liao, P. First principles study on hydrogen doping induced metal-to-insulator transition in rare earth nickelates RNiO3 (R = Pr, Nd, Sm, Eu, Gd, Tb, Dy, Yb). *Physical Chemistry Chemical Physics* **22**, 6888-6895, doi:10.1039/C9CP06522A (2020).

4 Michaelson, H. B. The work function of the elements and its periodicity. *Journal of Applied Physics* **48**, 4729-4733, doi:10.1063/1.323539 (1977).

5 Ren, H. *et al.* Controllable Strongly Electron-Correlated Properties of NdNiO3 Induced by Large-Area Protonation with Metal–Acid Treatment. *ACS Applied Electronic Materials* **4**, 3495-3502, doi:10.1021/acsaelm.2c00473 (2022).

6 Shi, J., Zhou, Y. & Ramanathan, S. Colossal resistance switching and band gap modulation in a perovskite nickelate by electron doping. *Nature Communications* **5**, 4860, doi:10.1038/ncomms5860 (2014).

7 Hauser, A. J. *et al.* Temperature-dependence of the Hall coefficient of NdNiO3 thin films. *Applied Physics Letters* **103**, 182105, doi:10.1063/1.4828557 (2013).

8 Ha, S. D. *et al.* Hall effect measurements on epitaxial SmNiO${}_{3}$ thin films and implications for antiferromagnetism. *Physical Review B* **87**, 125150, doi:10.1103/PhysRevB.87.125150 (2013).

9 Dey, T. *et al.* Kelvin Probe Force Microscopy Imaging of Plasticity in Hydrogenated Perovskite Nickelate Multilevel Neuromorphic Devices. *ACS Nano*, doi:10.1021/acsnano.4c11567 (2025).

10 Zhong, Y. *et al.* Dynamic memristor-based reservoir computing for high-efficiency temporal signal processing. *Nature Communications* **12**, 408, doi:10.1038/s41467-020-20692-1 (2021).

11 Milano, G. *et al.* In materia reservoir computing with a fully memristive architecture based on self-organizing nanowire networks. *Nature Materials* **21**, 195-202, doi:10.1038/s41563-021-01099-9 (2022).

12 Cucchi, M. *et al.* Reservoir computing with biocompatible organic electrochemical networks for brain-inspired biosignal classification. *Science Advances* **7**, eabh0693, doi:10.1126/sciadv.abh0693.

13 Moon, J. *et al.* Temporal data classification and forecasting using a memristor-based reservoir computing system. *Nature Electronics* **2**, 480-487, doi:10.1038/s41928-019-0313-3 (2019).

14 Zhong, Y. *et al.* A memristor-based analogue reservoir computing system for real-time and power-efficient signal processing. *Nature Electronics* **5**, 672-681, doi:10.1038/s41928-022-00838-3 (2022).
